# Letter from La Moille, Ill.

**Published:** 1882-02

**Authors:** Geo. J. Rice

**Affiliations:** LaMoille


### ARTICLE XIII.

LAMOILLE, Jan. 24, 1882.

EDITORS CHICAGO MEDICAL JOURNAL AND EXAMINER :—The order of our Board of Health, requiring the children of our public schools to be vaccinated, has been very thoroughly obeyed in some towns of our county, while others have almost entirely ignored it. As a consequence of the order, and of a case of small-pox in the autumn, we have had more vaccinating to do this winter than ever before in the same period of time. I have successfully vaccinated three hundred patients, using pure bovine virus in every case but two. It is in connection with the effects of this same bovine virus that I write this letter, as some of its results are somewhat peculiar and may be worthy of note in your paper. All the points used have been purchased of Sargent & Co., of Chicago. I vaccinated one hundred patients in November last, that run a typical course, with two exceptions, one a child not previously vaccinated, and the other a young lady previously vaccinated and having a typical mark. They each had four days of severe fever, accompanied by pains in back and limbs, soreness of eyeballs, etc., followed on the third day by a copious eruption of a dark purple color, intersected by streaks of sound skin of a yellowish white shade. The eruption was in some respects like that of rötheln, but much darker in shade. It lasted about four days, then peeled off without desquamation ; the arms at the same time were intensely swollen and almost black in color, with numerous yellow vesicles. I felt some concern at first, but they yielded to mild treatment, and were especially relieved by enveloping the swollen arm in vasaline dressing, recovering with an ulcer at the point of vaccination which was somewhat obstinate to heal.

Since the coming on of cold weather such cases have increased to an unpleasant extent; for the last fifteen days at least one in ten presenting the conditions described, only in a more exaggerated form—the fever being most intense and the eruption being more confluent in character. I neglected to say that eruption in all cases has been confined to parts of body and limbs covered with clothing, none appearing upon the face and but little upon the hands. So far all the cases have yielded to a covering of the arm affected with a dressing which excludes the air, a little alterative and laxative medicine. No serious consequences have resulted as yet, but the severity of its action is creating some alarm and a wide-spreading objection to vaccination, and some concern on my own part, for fear I may get some serious complications on my hands. I am not informed as to whether other practitioners are having the same trouble. GEO. J. RICE, M.D.

---

THE DE QUINCY HOME FOR INEBRIATES, conducted by Drs. H. H. Kane and W. H. Vittum, has been removed to Fort Washington, New York City.

THE unusual amount of sickness in this city during the last few weeks, and the extraordinary demands which have, as a consequence, been made upon the members of the medical profession, have taxed severely many practitioners. Among those who are now ill, may be named Dr. E. O. F. Roler, who has been compelled to go to the South for a brief respite from his arduous duties as an active obstetrician; Dr. D. A. K. Steele, of the new Medical School, who is now recovering from a severe prostration; Dr. W. S. Haines, who has also been obliged to temporarily absent himself from his active work in the chemical laboratory of Rush Medical College; and Dr. Alexander Fisher, who, though compelled more than a year ago to abandon his professional career in consequence of the infirmities of age, is now lying seriously ill.

DURING the closing week of the past month, a veteran physician and pioneer in this Western metropolis, Dr. L, D. Boone, went to his long rest. Dr. Boone was a descendant of the famous Daniel Boone, of Kentucky.

